# Dimensionality and optimal combination of autonomic fear-conditioning measures in humans

**DOI:** 10.3758/s13428-024-02341-3

**Published:** 2024-02-29

**Authors:** Federico Mancinelli, Juliana K. Sporrer, Vladislav Myrov, Filip Melinscak, Josua Zimmermann, Huaiyu Liu, Dominik R. Bach

**Affiliations:** 1 University of Bonn, Transdisciplinary Research Area “Life and Health”, Hertz Chair for Artificial Intelligence and Neuroscience, Bonn, Germany; 2 Wellcome Centre for Human Neuroimaging, University College London, London, UK; 3 Department of Psychiatry, Psychotherapy, and Psychosomatics, University of Zurich, Zurich, Switzerland; 4 Department of Neuroscience and Biomedical Engineering, Aalto University, Espoo, Finland; 5 Department of Cognition, Emotion, and Methods in Psychology, University of Vienna, Vienna, Austria

**Keywords:** Fear conditioning, Psychophysiological modeling, Threat conditioning measures, Inter-individual variability, Mega-analytic, Conditioned autonomic responses, Optimal combination

## Abstract

Fear conditioning, also termed threat conditioning, is a commonly used learning model with clinical relevance. Quantification of threat conditioning in humans often relies on conditioned autonomic responses such as skin conductance responses (SCR), pupil size responses (PSR), heart period responses (HPR), or respiration amplitude responses (RAR), which are usually analyzed separately. Here, we investigate whether inter-individual variability in differential conditioned responses, averaged across acquisition, exhibits a multi-dimensional structure, and the extent to which their linear combination could enhance the precision of inference on whether threat conditioning has occurred. In a mega-analytic approach, we re-analyze nine data sets including 256 individuals, acquired by the group of the last author, using standard routines in the framework of psychophysiological modeling (PsPM). Our analysis revealed systematic differences in effect size between measures across datasets, but no evidence for a multidimensional structure across various combinations of measures. We derive the statistically optimal weights for combining the four measures and subsets thereof, and we provide out-of-sample performance metrics for these weights, accompanied by bias-corrected confidence intervals. We show that to achieve the same statistical power, combining measures allows for a relevant reduction in sample size, which in a common scenario amounts to roughly 24%. To summarize, we demonstrate a one-dimensional structure of threat conditioning measures, systematic differences in effect size between measures, and provide weights for their optimal linear combination in terms of maximal retrodictive validity.

## Introduction

Pavlovian fear conditioning, more recently also termed threat conditioning (LeDoux, 2014), is a laboratory model of aversive associative learning with translational value in the development of clinical interventions for anxiety disorders (VanElzakker et al., 2014; Bach et al., 2018; Fullana et al., 2020; Beckers et al., 2023). In this paradigm, a conditioned stimulus (CS+) is contingently coupled with an aversive unconditioned stimulus (US), and a different CS− is never paired with the US. In humans, threat conditioning is commonly inferred from a difference between conditioned autonomic nervous system (ANS) responses to CS+ and CS− (Ojala & Bach, 2020; Lonsdorf et al., 2017). This includes, among others, skin conductance responses (SCR; Boucsein, 2012; Bach et al., 2010; Staib et al., 2015, pupil size responses (PSR; Korn et al., 2017), heart period responses (HPR; Castegnetti et al., 2016) and respiratory amplitude responses (RAR; Castegnetti et al., 2017). In all of these measures, a CS+/CS− difference is consistently observed during threat conditioning, and in recall tests after consolidation. However, our understanding of these measures still has at least two noteworthy gaps to address: their inherent dimensionality, i.e., the number of underlying factors, and the potential for their combined utilization to enhance precision in inferring threat conditioning (Bach et al., 2018).

Crucially, the physiological processes by which these conditioned responses are elicited diverge. The electrical conductance of the skin rises as a result of sweating but diminishes when the sweat evaporates. The opening of these eccrine sweat glands is triggered by the activation of sympathetic sudomotor nerve fibers (Boucsein, 2012; Gerster et al., 2018). SCR elicited during CS+ presentation are higher than those during CS− (see, e.g. Boucsein, 2012). Changes in pupil size are due to sympathetic innervation, which dilates the pupil and parasympathetic innervation, which constricts the pupil (Loewenfeld & Lowenstein, 1999; McDougal & Gamlin, 2008). A CS−related dilation of the pupil is a well-established phenomenon (Korn et al., 2017; Leuchs et al., 2017; Reinhard & Lachnit, 2002; Reinhard et al., 2006; Visser et al., 2013, 2015, 2016). The cardiovascular system is innervated by both sympathetic and parasympathetic branches of the autonomic nervous system, which control heart period (the reciprocal of heart rate; Berntson et al., 2007). Many studies have shown that threat-predictive CS results in bradycardia, i.e. an increase in heart period, with the fast time course suggesting a predominantly parasympathetic influence (see Castegnetti et al., 2016, for a review of studies). Breathing is unique in that it is primarily regulated by the ANS but can also be influenced voluntarily (Barnes, 1986; Hlastala & Berger, 2001; Kreibig, 2010; Lorig, 2007). Although breathing patterns have been less commonly explored in threat-conditioning experiments, a few studies have demonstrated that threat-predictive CS induce a decrease and later increase in respiration amplitude (Castegnetti et al., 2017; Van Diest et al., 2009). Taken together, these observations suggest that threat-conditioned SCR and PSR are predominantly under sympathetic influence, HPR is under parasympathetic influence, and RAR is under both. Here, we examine CS+/CS− differences in conditioned SCR, HPR, PSR, and RAR, averaged over all trials of an acquisition session (Lonsdorf et al., 2017). There are several possible scenarios for the underlying structure of the resulting measures. In the simplest case, one might assume the existence of a single underlying (latent) CS-US association, between-person variation in this latent association, and a fixed mapping (e.g., scaling) from this association onto conditioned responses. With independent observation noise, the ensuing measures will vary systematically only along one dimension. Different from this situation, the mapping from latent association to conditioned responses could be systematically different between subsets of measures, for example, between predominantly sympathetic and parasympathetic responses. Between-person variability in these two scaling factors could then result in a two-factorial structure. Next, there is a possibility that observation noise in the ANS measures co-varies. For example, a voluntary (i.e., not threat-conditioned) deep breath affects the measured respiration response but also the heart period response (via respiratory arrhythmia, mediated by a mechanical influence on the vagal nerve). Finally, it has been suggested that different autonomic threat-learning measures may relate to different quantities in the learning process (Ojala & Bach, 2020), based on trial-by-trial learning trajectories (Li et al., 2011; Zhang et al., 2016; Tzovara et al., 2018; Homan et al., 2019) or pharmacological interventions (Bach et al., 2018). Inter-individual differences in these different learning quantities could again affect the inherent structure of autonomic measures. Noteworthy, for us to be able to capture any of these scenarios, meaningful between-person variability in the learning process is crucial.

We further explored the potential benefits of combining multiple measures to enhance the accuracy of inference about whether learning has occurred, a common question for example in preclinical intervention research (Bach et al., 2018). Under baseline conditions, the effect size to distinguish CS+ and CS− (i.e., retrodictive validity) can be taken as a metric for the accuracy of this inference (Bach et al., 2020, 2023) and can be quantified, for example, by Cohen’s *d*. Thus, for each measure, its effect size is fully determined by its mean and variance across participants. If we combine measures linearly, the weight of each measure will reflect the relative balance of the mean of the CS+/CS− difference for this measure, and its variability. Some measures may for instance exhibit high differences across CS+/CS− conditions, but with substantial variability, while others may demonstrate more modest, yet consistent, differences. These variations could stem from factors such as observation noise or inherent physiological variability. Here, we provide an empirical analysis of the benefits of combining conditioned responses, and provide a quantitative metric of the generalizability of this approach, which might help planning future threat conditioning studies.

To summarize, the goal of this study was to (1) determine the dimensionality of between-person variance in four different conditioned responses including those under sympathetic and parasympathetic influences (SCR, PSR, HPR, RAR), and (2) to give a quantitative assessment of the improvement in performance when these are combined.

**Table 1:** Overview and demographics for the nine included data sets

Not all data sets included all conditioned responses. Initial *N* refers to the number of participants who completed the study per protocol, final *N* to the number of participants included in the analysis after data quality control. When there are two rows for a dataset, the first refers to SCR data and the second to PSR data. Demographics (sex, age), percentage of incorrect data (% incorr), and percentage of missing data (% miss; PSR: eyeblinks, saccades, loss of fixation; SCR: artefacts) refer to the final sample. The percentage of missing data corresponds to the mean percentage of missing values in the indices per condition. No data exclusion was applied to HPR and RAR. See 2.4. ‘Data preprocessing’, for modality-specific details

## Method

### Participants

We re-analyzed nine threat-conditioning datasets which comprised a total of 256 individuals. Eight of these data sets were included in previous publications: PubFe (Korn et al., 2021), SC4B (Staib et al., 2021), VC7B (Staib et al., 2021b), DoxMemP (Khemka et al., 2021), FR (Tzovara et al., 2021), TC (Tzovara et al., 2021), FSS6B (Staib et al., 2021a), FER02 (Zimmermann et al., 2021). One experiment (FER01) is first published here (see Table 1 for details). The datasets used in this study are publicly available on Zenodo and can be browsed through the community “PsPM development data” (https://zenodo.org/communities/pspm/). The reference section of this article includes URLs to all the datasets. Pre-processed data, including all participant- and condition-wise response estimates, are available on OSF (https://osf.io/cmaq7/), under ‘Learning indices/Conditions’, and the corresponding pre-processing code under “Analyses/Pre-processing”. An R markup file containing this analysis (i.e., ‘combining-measures.Rmd’) can be found on the same webpage, under ‘Analyses/Combining measures’. All experiments included unique, healthy, unmedicated individuals, recruited from the student and general population. Participants confirmed that they had no history of neurological, psychiatric, or systemic medical disorders, and all had normal or corrected-to-normal vision. Each study, including the form of taking written informed consent, was conducted in accordance with the Declaration of Helsinki and approved by the governmental research ethics committee (Kantonale Ethikkommission Zurich, KEK-ZH-2013-0118).

Participant exclusion criteria centered on both the absence of a substantial portion of data, and considerations related to learning outcomes. First, we excluded participants who had more than 50% missing pupil size data over the time interval from trial onset to the next trial onset, in over 50% of the trials, or experienced extended detachment of SCR electrodes. Further, we excluded all RAR measurements from dataset FER01 due to the implausibly low registered effect size ($-0.02$, as opposed to the average of $-0.41$ from the remaining datasets) which indicate systematic technical problems. Finally, we excluded three participants altogether on account of their implausible conditioned response differences: (1) dataset FER02, HPR = $-162.7$, more than 5 standard deviations from mean across all datasets; (2) dataset FER02, SCR= $-0.40$, more than 3 standard deviations from mean across all datasets; (3) dataset SC4B, SCR= $+0.75$, more than 3 standard deviations from mean across all datasets. See Table 1 for a summary of excluded participants for each data set.

### Stimuli and procedure

All but one experiment (TC) implemented delay threat conditioning with a 4-s CS, a 0.5-s US, and CS-US onset interval of 3.5 s (i.e., co-termination of CS/US). TC used short-interval trace threat conditioning with a 3-s CS and a 1-s trace interval, resulting in a CS-US onset interval of 4 s. Inter-trial interval was randomly determined on each trial to be 7, 8, 9, 10, or 11 s in FER01/02, and 7, 9, or 11 s for the other experiments. The reinforcement schedule of CS/US was 50%. CS were visual, auditory or somatosensory, and summarized in Table 1. US was a train of electric square pulses delivered with a constant current stimulator (Digitimer DS7A, Digitimer, Welwyn Garden City, UK). US intensity was set individually for each participant to an unpleasant but not painful level using an ascending staircase until stimuli were clearly painful, followed by delivery of 14 random stimuli below this upper limit. The final stimulus intensity was set as  85% of the intensity rated by participants as clearly painful.

Several experiments used two CS+ (FER01/02, SC4B, FSS6B, VC7B) and some of these used two CS− (SC4B, FSS6B, VC7B). We averaged over both CS of the same type, under the assumption that learning is not different between the two CS sets, and that averaging will simply increase the signal-to-noise ratio for all measures equally, thus not affecting their dimensionality or most discriminant combination. We note that for FER01/02, both CS+ were of the same type (triangles of different colors). For SC4B/FSS6B/VC7B, where they were of different types within the same sensory modality, there was no indication of different learning for the two qualitatively dissimilar CS sets. For experiments with an incidental task of indicating CS (physical) identity on each trial (FER02, PubFe, SC4B, DoxMemP, FSS6B, VC7B), trials with an incorrect response were excluded from the analysis.

### Data recording

All experiments took place in a soundproof chamber. The same recording systems were used for all studies. Pupil diameter and gaze direction were recorded using an EyeLink 1000 System (SR Research, Ottawa, ON, Canada) at a sampling rate of 500 Hz. Calibration of gaze direction was performed with the nine-point calibration protocol implemented in the EyeLink 1000 software. Participants placed their heads on a chin rest at a distance of 70 cm in front of the monitor (Dell P2012H, 20” set to an aspect ratio of 5:4, 60-Hz refresh rate).

The output signal of all other physiological measures was digitized at a sampling rate of 1000 Hz using a DI-149 AD converter (Dataq Inc., Akron, OH, USA) and recorded with Windaq (Dataq Inc.) software.

Skin conductance electrodes were placed on the thenar/hy-pothenar of the left hand for FER01/02, and the non-dominant hand for all other data sets. We used 8-mm Ag/AgCl cup electrodes (EL258, Biopac Systems Inc., Goleta, CA, USA) and 0.5% NaCl gel (GEL101, Biopac Systems Inc., Goleta, CA, USA; Hygge and Hugdahl, 1985). Skin conductance signal was amplified with a SCR coupler/amplifier (V71-23, Coulbourn Instruments, Whitehall, PA, USA).

ECG was recorded with four 45-mm, pre-gelled Ag/AgCl adhesive electrodes attached to the four limbs. The experimenter visually identified the lead (I, II, III) or the augmented lead (aVR, aVL, aVF) configuration that displayed the highest R spike and only recorded this configuration. Data were pre-amplified and 50-Hz notch-filtered with a Coulbourn isolated five-lead amplifier (LabLinc V75-11, Coulbourn Instruments, Whitehall, PA).

Respiratory time series were collected with an aneroid chest bellows (V94-19, Coulbourn Instruments, Whitehall, PA, USA) and differential aneroid pressure transducer (V94-15, Coulbourn) fitted around the rib cage over the lower end of the sternum. The signal was amplified using a resistive bridge strain gauge transducer coupler (V72-25B Coulbourn).

### Data preprocessing

For data pre-processing and parameter extraction, we used MATLAB (Version R2019a, MathWorks, Natick, MA, USA) and PsPM (Psychophysiological Modeling, https://bachlab.github.io/PsPM/, Version 5.1.1), a MATLAB toolbox for model-based analysis of psychophysiological data (Bach & Friston, 2013; Bach et al., 2018).

SCR artefacts were detected via an initial automatic quality assessment excluding data outside of the normal range of 0.05-60  $\upmu$S or with a slope higher than 10 $\upmu$Ss^-1^. Subsequently, SCR data were visually examined, a process which included rejection/confirmation of detected artefacts, and detection of additional artefacts. Those artefacts were marked, and if they were shorter than 2 s, then corresponding data points were linearly interpolated for filtering and excluded for model inversion. For longer artefacts, the remaining data intervals were separately filtered and analyzed. We filtered SCR data (first-order bidirectional band-pass Butterworth filter, 0.0159-5 Hz) and downsampled to 10 Hz (Bach et al., 2010; Staib et al., 2015). To estimate the amplitudes of anticipatory SCR, we used a constrained dynamic causal model (DCM) with fixed dispersion but flexible latency for the anticipatory response during CS, and fixed dispersion/latency for the US- or US omission-evoked response, as implemented in PsPM (Bach et al., 2010; Staib et al., 2015). This approach estimates sudomotor nerve (SN) activity, given observed changes in skin conductance, under a linear time-invariant model of the SN-SCR relationship (Bach et al., 2010) and provides trial-by-trial estimates of the conditioned response amplitude (Bach et al., 2018). These were then averaged within each participant and condition.

The EyeLink 1000 System uses an online parsing algorithm to detect saccades and eye blinks, which were excluded. Preprocessing followed the procedure by Kret and Sjak-Shie (2019) as implemented in PsPM 5.1.1. This procedure identifies valid samples by range, speed, edge, trendline, and isolated sample filtering. The data of the two eyes were averaged if they were both recorded and missing data points were linearly interpolated. Pupil data were filtered (lowpass Butterworth filter, cut off 50 Hz) and downsampled to 100 Hz. Finally, pupil size data for which combined gaze direction was outside ± $5^{\circ }$ visual angles around the fixation points were treated as missing data points and were excluded for analysis as in previous work (Korn et al., 2017). To estimate the conditioned pupil response amplitude on a condition-by-condition level, we used the general linear convolution model (GLM) implemented in PsPM and developed by Korn and Bach (2016).

QRS complexes were detected from ECG data using a modified Pan and Tompkins algorithm (Paulus et al., 2016) to create heartbeat time stamps. These were transformed into an interpolated heart period signal specifying an upper and lower limit for heart periods of 0.4 and 1.2 s respectively; with an interpolation sampling rate of 100 Hz. Then, the heart period time series were band-pass filtered with a bidirectional Butterworth filter (0.015-0.5 Hz) and down-sampled to 10 Hz, we used the default GLM implementation in PsPM to estimate the amplitude of conditioned HPR (Castegnetti et al., 2016).

Raw respiratory traces were converted to interpolated respiration amplitude time series with a previously published respiratory cycle detection algorithm and a 10-Hz sampling rate (Bach et al., 2016). After the respiration amplitude time series were band-pass filtered with a bidirectional Butterworth filter (0.01-2 Hz), we estimated the amplitude of RAR with the default GLM implemented in PsPM (Castegnetti et al., 2017).

Due to our method of interpolating data and the use of a convolution model, responses to the US can affect the response even before the US occurs. This is why we excluded reinforced trials (i.e., when US is present) from all statistical analyses.

### Statistical analyses

A publicly accessible R MarkDown document containing our statistical analyses can be found on OSF (https://osf.io/cmaq7/, under “Analyses/Combining measures”). Our analyses focus on the CS+/CS− ANS measurement difference for each participant. We scaled the measurements by dividing the differences by the standard deviation of the originating dataset, to account for potential trivial differences in the scaling of the measurement system. The effect size for CS+/CS− differences was expressed as Cohen’s *d*.

#### Dimensionality of ANS measures

We conducted an exploratory factor analysis (EFA) to probe the latent dimensionality of the between-person variance of our measures. This analysis included participants from two sources: (1) the largest single dataset FER02, which encompasses all measures, and (2) all datasets. FER02 offers homogenous data, but has limited sample size. On the other hand, incorporating participants from multiple datasets provides a much larger sample size, but may suffer from systematic differences in ANS measures (e.g., owing to task peculiarities, such as the different types of CS, or experimenter differences). This analysis was carried out using the full range of measures available as well as subsets of three, as larger samples were available for some subsets of measures. We used the ‘fa’ function from R package *psych*, version 2.2.9, and the ‘paran’ function from package *paran*, version 1.5.2, as well as custom R code to compute empirical *p* values. The factors yielded by the procedure were left unrotated. Our parallel analysis approach retained components whose eigenvalues were larger than in randomized data at a significance level of $p \le 0.05$ (Glorfeld, 1995).

**Table 2:** Parallel analysis results for the FER02 dataset and for the combined dataset

	

For the combined dataset, we also show results for all subsets of three measures. The gray cells indicate the presence of the respective measure in the analysis. The empirical *p* values are calculated through a Monte Carlo simulation: the process involves counting the number of instances in which the original eigenvalues were found to be lower than those obtained from factor analysis on a randomized data matrix in 5000 simulations. The sample size for each analysis is reported in the last column. The first row shows the results from the FER02 dataset, and the other rows different combinations of measures from all datasets

#### Combining ANS measures optimally

We investigated whether a linear combination of measures could achieve a higher Cohen’s *d* than the best-performing measure for a given dataset. The optimal weights can be obtained analytically by maximizing the quantity *d*, i.e., Cohen’s *d* for combinations of measures.

Consider a vector of weights $\textbf{w}$ and the vector of measurements $\textbf{x}_i$ for each of *S* participants, indexed by *i*. The combined measure for participant *i* is given by $\textbf{w}^\text {t} \textbf{x}_i$. The empirical mean and standard deviation of this measure across participants are given by $\mu = \textbf{w}^\text {t} \textbf{m}$ and $\sigma = \sqrt{\textbf{w}^\text {t} \Sigma \textbf{w}}$, respectively, where $\textbf{m} = \frac{1}{S} \sum _S \textbf{x}_i$ is the mean measurement vector and $\Sigma = \frac{1}{S-1} \sum _S (\textbf{x}_i - \textbf{m}) (\textbf{x}_i - \textbf{m})^\text {t}$ is the covariance matrix of the $\textbf{x}_i$’s.

To maximize *d*, we can equivalently maximize $d^2 = \frac{\textbf{w}^\text {t} \textbf{m} \textbf{m}^\text {t} \textbf{w}}{\textbf{w}^\text {t} \Sigma \textbf{w}}$, which is the objective function of Fisher’s linear discriminant analysis (LDA). This equivalence arises from the fact that maximizing Cohen’s *d* is the same as maximizing the ratio of the between- and within-class covariance matrices of two classes: one which includes the set of measurement differences, and the other consisting solely of the origin. Optimal weights for LDA can be found in multiple ways, with the solution $\textbf{w}^* \propto \Sigma ^{-1} \textbf{m}$ (see Bishop, 2006, for a brief derivation). To give a geometric sense of the result, note that the weights are oriented towards the direction of the mean of measurement differences, which prioritizes measurements that are highly discriminating; multiplying by the precision matrix $\Sigma ^{-1}$ rotates the mean vector to maximize precision, penalizing directions in which measurement differences are more variable.

Our analyses drew participants from all datasets, and considered all subsets of 2–4 measures, to offer some insight as to which measures are most effective in different combinations. Note that the datasets used in this study were not originally collected with the specific objectives of our current paper in mind. Consequently, there are variations in the number of recorded measures between datasets (Table 1). These differences resulted in variations in sample sizes when merging the datasets according to all subsets of measures (Tables 2 and 4). Our analyses further ramified in terms of the way we obtained and tested optimal weights. Specifically, we report on performance both (1) in-sample, i.e., using the same dataset to find optimal weights and comparing the ensuing combined measure to the best individual measure - but also (2) out-of-sample (OOS), testing how the optimal weights found in a (training) portion of data might generalize when deployed on the leftover (test) portion of data. The two approaches are complementary. The first allows us to draw inferences about the highest effect size that can be achieved by combining measures within a dataset, while enabling us to infer the corresponding weights. On the other hand, the second approach probes the capability of these optimal weights to generalize beyond the given dataset. It yields estimates for the expected performance of the derived weights when applied to new data, as well as measures of uncertainty associated with those estimates. To compute the average OOS performance and the uncertainty estimates around it (which we report as 90% confidence intervals) we used the package *nestedcv* (github.com/stephenbates19/nestedcv), which applies a correction to the (biased) uncertainty estimates which arise from conventional repeated cross-validation (Bates et al., 2021). Specifically, our procedure utilized five-fold cross-validation over 1000 repetitions. In each repetition, four of the folds were used as the training set, while the leftover fold was designated as the test set. These folds were pseudo-randomly sampled. The full pseudocode outlining the procedure, which we have used in its R implementation (found at github.com/stephenbates19/nestedcv), can be found on p. 15, Algorithm 1, in Bates et al. (2021).

**Table 3:** Cohen’s *d* for individual datasets and measures, and their combination using weights derived in-sample

Dataset	Individual measures				Best	Optimal	Gain	Sample size
	(Cohen’s *d*)				measure	combination	(Cohen’s *d*)	
	SCR	PSR	RAR	HPR	(Cohen’s *d*)	(Cohen’s *d*)		
DoxMemP	0.81	$\cdot$	0.55	1.26	1.26	1.66	$+0.40$	20
FER01	0.40	0.37	$\cdot$	0.74	0.74	0.89	$+0.16$	26
FER02	0.40	0.52	0.28	0.26	0.52	0.65	$+0.13$	68
FR	0.74	$\cdot$	0.52	1	1	1.03	$+0.03$	22
FSS6B	0.44	0.49	$\cdot$	$\cdot$	0.49	0.57	$+0.08$	17
PubFe	0.44	0.93	0.35	0.74	0.93	0.99	$$ +0.06$$	12
SC4B	0.75	1.08	0.22	0.77	1.08	1.85	$$+0.77 $$	8
TC	0.70	$$\cdot $$	0.55	1.20	1.20	1.21	$$ +0.01$$	19
VC7B	0.77	0.66	$\cdot$	$\cdot$	0.77	0.83	$+0.06$	17

Individual measures: the *d* values for all available measures of the corresponding dataset. A *dot* represents a measure’s unavailability. Best measure: measure with highest *d*. Optimal combination: summarizes the *d* from the optimal (linearly combined) measure; optimal weights were computed as per Eq. (2). Gain: summarizes the difference between the combined measure’s Cohen’s *d* and the measure achieving the highest effect within the dataset. Finally, the last column individuates the sample size for each dataset

**Table 4:** In-sample results for the combined dataset

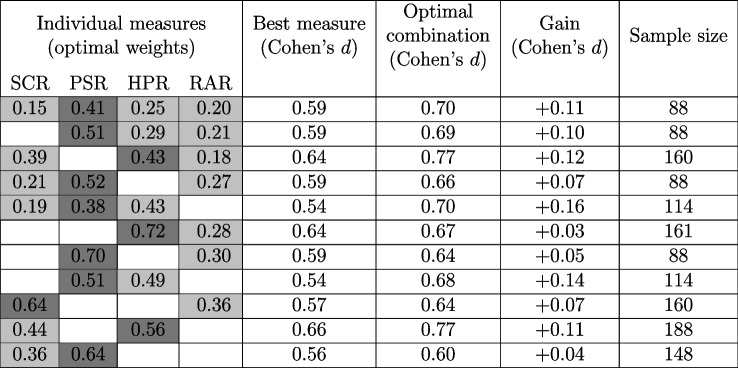	

Columns 1–4 are color-coded to indicate the presence and effect size of different measures used in the corresponding analysis (*white*: measure absent, *gray*: measure present, *dark gray*: measure with highest Cohen’s *d*). Within each cell, we report the weight taken for the combined measure, divided by the 2-norm of the weight vector. Best measure: Cohen’s *d* for the best individual measure, indicated by *dark gray* on the left-hand side). Optimal combination: Cohen’s *d* for the combined measure. Gain: increase in effect size achieved by using the combined measure instead of the best measure. *N*: sample size for each analysis

## Results

### Dimensionality of ANS measures

We found no evidence for more than one factor underlying between-person variability in the CS+/CS− difference (see Table 2). Factor loadings largely reflected the effect sizes of each ANS measure to distinguish CS+/CS−. Loadings for factor analysis on all measures for FER02, and for combined datasets, were largely in agreement, with the highest loading from PSR (FER02: 0.67; all data: 0.73), SCR (FER02: 0.61 ; all data: 0.59), HPR (FER02: 0.34; all data: 0.41), and lastly RAR (FER02: 0.07; all data: 0.10).

### Combining ANS measures optimally

#### In-sample

Table 3 shows results for all individual datasets separately, with all available measures and their optimal combination using weights derived in-sample. Table 4 includes the results of our analyses in the merged dataset. Combining measures yielded clear-cut improvements. The optimal weights favor ANS measures with high CS+/CS− difference, adjusting by their precision so that less reliable measures are penalized (the normalized weights are reported in Table 4). On average, the combined measure allowed for a gain in Cohen’s *d* of +0.09 ± 0.04 (mean ± standard deviation), without ever causing a decrease compared to the best single measure. It is important to note that this is only a best-case scenario because the weights are obtained using the same data on which they are ultimately tested. As such, the increases in effect size registered in Table 4 should be regarded as an upper limit, and should only be used to obtain a lower bound for the number of participants required. Thus, while the weights shown can be used to combine measures outside our data, one should refer to the *expected* out-of-sample performances in the next section when performing power calculations.

**Table 5:** Out-of-sample results for the combined dataset

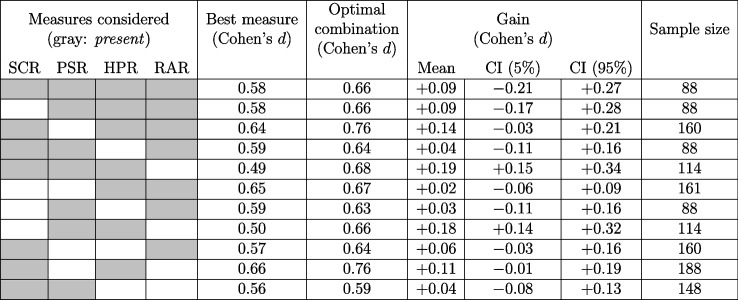	

Columns 1–4 are color-coded to indicate the presence and effect size of different measures used in the corresponding analysis (*white*: measure absent, *gray*: measure present). No single best measure is indicated since this depends on the training data set on each iteration. Single best measure: average OOS Cohen’s *d* for the best single measure in each training iteration; Optimal combination: the average (across training-test set combinations), OOS, Cohen’s *d* effect achieved by the optimal combination of measures. Gain Mean: average difference in effect size between combined measures and best measure, and bias-corrected 90% confidence intervals, from 5% (column 8), to 95% (column 9)

#### Out-of-sample

Table 5 displays the results from our out-of-sample analyses. The actual sample size for each combination of measures is reported in the table. This analysis shows an increase in average performance (mean ± s.d., across all subsets of measures: 0.09 ± 0.06), of similar size as in the in-sample setting. The corrected confidence intervals differ between combinations due to the different sample size.

To provide a sense of the gains from utilizing the combined measure, we can compute a power analysis for a hypothetical threat-learning study. Based on the out-of-sample results, we would expect that the best single measure has an effect size of 0.58, and the combined measure an effect size of 0.66. If we were to aim for a power of 80% (at a two-tailed significance level of 0.05) to detect such effects in a one-sample *t* test, we would on average need *N* = 25 participants utilizing the single best measure, whereas utilizing the combined measure would only require *N* = 19. Of course these are only general considerations, since the actual benefit will depend on the measures. Noteworthy, PSR and HPR measures appear to benefit particularly from their combination, showing confidence intervals well above zero, both when they are combined with SCR, and among one another.

## Discussion

To quantify threat learning, several conditioned responses are conventionally deployed to infer on the latent CS-US association. This plurality poses a challenge for researchers, who often have to settle on one individual measure in order to avoid correction for multiple comparisons, or to pre-register a single primary analysis and outcome. Selecting a suitable measure is far from trivial, as these measures not only vary in their sensitivity, but may also reflect distinct underlying neural or psychological processes (Ojala & Bach, 2020). Here, we investigated the dimensionality of threat learning measures, and their optimal combination, in a large sample of participants.

We found no evidence for more than one underlying factor across the full range of four measures or in subsets of three measures. This suggests that, despite potential heterogeneity in the underlying learning quantities (and/or neural systems), the inter-individual variability in experiment-averaged threat learning measures largely stems from a single source. Further work, possibly availing of hypothesis-driven confirmatory factor analysis approaches, and a suitable (Bayesian) framework, could provide conclusive evidence for this one-dimensional structure. It is, however, possible that including trial-wise measurements, in conjunction with structural equation modeling, might uncover more sophisticated factor structures. For instance, there might be factors for predominantly sympathetic (e.g., SCR) or parasympathetic (e.g., HPR) measures, or discriminate slow (e.g., RAR) and fast (e.g., PSR) sub-systems that control anticipatory responses to aversive outcomes. Further, our focus was solely on autonomic measures. Including other conditioned responses, such as fear-potentiated startle, or explicit CS-US contingency ratings, may diverge from the one-dimensional structure identified in this study.

Next, we were able to quantify the extent to which an optimal combination of measures yields a higher effect size than the best-discriminating individual measure. We quantified the precise gain achieved by all possible combinations of measures, in settings that differed in the way that the optimal weights were derived and tested (either in- or out-of-sample). We observed similar improvements in either scenario, which were meaningful, albeit modest, in size (+0.09 in Cohen’s *d*). We emphasize, however, that in the in-sample setting, the combined measure only provides an upper-bound of the effect size, as it is inherently biased: the combination is indeed constructed to achieve a higher Cohen’s *d* than any individual measure, albeit of course the actual increase in effect size will still depend on the measures at play. Our in-sample results are therefore only relevant when performing best-case power analyses (i.e., when the quantity of interest is the maximal Cohen’s *d*). Importantly, our out-of-sample analyses complement these weights by providing a measure for their capacity to generalize. The results of these latter analyses can thus be referred to when performing power analyses, which involve *expected* effect sizes. In the out-of-sample setting, the gains in Cohen’s *d* were conspicuous, indicating good generalizability of weights. Caution should be exercised with regard to the confidence intervals (which depend on various factors, such as sample sizes) and the actual measures involved. Indeed, on close inspection, it becomes salient that certain combinations of measures exhibit a more substantial improvement. For instance, combining PSR and HPR appears to cause noticeable improvements, both when the two are considered in isolation, or indeed as we add further measures. This might arise as PSR and HPR measurements constitute more independent (or less redundant) sources of information than other measurement pairs about the latent CS-US association. Finally, the optimal weights derived here can be used outside of our data, where we would suggest using the weights derived in-sample (as they are based on a large number of participants).

In sum, we provided evidence that optimally combining measures can serve as a valuable tool for researchers to refine their methods. Our findings, based on the specific paradigm used (threat acquisition with a 3.5-s CS-US latency), may provide a foundation for extending this approach to various other phases of conditioning paradigms, such as reactivation and extinction, although, of course, empirical validation of this hypothesis is necessary. The weights obtained here for combined datasets (e.g., those outlined in Table 4) were based on a large number of participants (ranging from 88 when considering all measures, to 188 when only considering SCR and HPR), and thus can be used verbatim for threat conditioning measures based on psychophysiological modeling (Bach and Friston, 2013; Bach et al., 2018), and in experimental settings similar to the ones reported here, since data homogeneity was not a crucial issue for the improvements in effect size. The formula for obtaining the weights holds for any combination of measurements and can easily be extended to entirely different measures. In particular, threat- conditioning studies sometimes test recall after an intervention targeted to impair synaptic consolidation (e.g., Bach et al., 2018; Kindt et al., 2009; Wehrli et al., 2023). It would be useful to extend our current results on threat learning to recall tests.

Complementary to our main findings, our analyses confirm the previous notion that PSR has the highest effect size when compared to SCR, HPR, or RAR (Korn et al., 2017), while HPR and SCR were comparable (Castegnetti et al., 2016). RAR appeared to be the least discriminative measure, albeit with large variability across individual studies. It has been suggested that to robustly quantify respiration amplitude, a double-belt system is required (Binks et al., 2006). While we have demonstrated across several experiments that single-belt systems do allow inference on cognitive processes (Bach et al., 2016; Castegnetti et al., 2017), it is likely that the precision of this inference depends on the precise positioning of the belt and of the participant, which are substantive sources of variability across experimenters and setups. Our conclusions regarding RAR should therefore be treated cautiously, simply as they may not extend to other laboratory conditions or belt systems. It is worth noting that respiration is among the less commonly utilized measures, and further research is warranted to fully explore its potential (Ojala & Bach, 2020).

In our mega-analytic approach, we combined various data sets with only slight discrepancies in experimental set-ups (e.g., number of CS+’s, length of inter-trial intervals, and similar) that are inconsequential for the specific objectives of our analyses. Inspection of the individual effect sizes revealed a systematic variation, across all autonomic measures, between experiments. The choice of data sets was driven by their public availability and similarity of setups, rather than being optimized to investigate underlying reasons. We emphasize that our results were robust across different (overlapping) combinations of data sets, and do not appear to be exclusively driven by one or a small number of experiments in our sample.

Some caveats and limitations merit attention in our study. Firstly, we should note that while our aggregated data is in a sense heterogeneous (in terms of the experimental setups, experimenters involved, CS modalities, and so on) it does all come from one single laboratory which used relatively similar experimental structures and recording equipment. To fully delineate the benefits of combining measures in truly heterogeneous settings, it would be useful to extend our analyses to setups utilized by wholly different laboratories. However, currently, there is a dearth of human threat-conditioning studies reporting several autonomic measures at the same time (see Leuchs et al., 2019, for a notable exception). To ensure robustness across laboratories, the current results would ideally be reproduced in a multi-lab calibration experiment, as has recently been proposed (Bach et al., 2023).

Further, our analyses are based on measurement averages over acquisition, and so might not generalize to trial-by-trial response quantification. Firstly, averaging over acquisition might have dissipated subtle differences that could have been observed by looking at specific phases of the task – such as focusing exclusively on the initial trials. This consideration is pertinent to both our dimensionality reduction, and optimal combination, results. Driven by appropriate hypotheses about the exact segments of the task to be considered, selectively examining subsets of trials is an interesting avenue for further research. Secondly, by linearly combining measures according to the weights we derived, it is plausible to enhance the power for assessing individual variations, in terms of their relationship (e.g., correlation) with task-based metrics. However, because our method primarily examines task-averaged quantities, it likely will not be immediately beneficial for looking at the fine-grained temporal evolution of responses. Thus, when studying task-based behavior, it is critical to recognize that examining the temporal progression of the combined measure may not yield significant improvements since, again, the optimal weights were determined based on time-averaged data.

To summarize, we provide evidence that between-person variability in threat-conditioned responses, averaged over trials, are underpinned by a single factor, and show that combining them yields a benefit in terms of retrodictive validity, i.e., the ability to distinguish CS+ and CS−. This benefit is not only theoretical (i.e., in-sample) but also practically relevant, as the optimal weights derived in a subset of participants generalize to the remaining participants. As such, the optimal weights we give here could be used for future studies. The OOS performance reported here (and relative, bias-corrected, confidence intervals) could constitute a valuable resource to experimenters as they face experimental cost–benefit considerations. Thus, we believe this work could complement ongoing efforts to optimize the accuracy of individual threat-learning measures.
