# Publisher Correction: Dimensionality and optimal combination of autonomic fear-conditioning measures in humans

**DOI:** 10.3758/s13428-024-02391-7

**Published:** 2024-03-22

**Authors:** Federico Mancinelli, Juliana K. Sporrer, Vladislav Myrov, Filip Melinscak, Josua Zimmermann, Huaiyu Liu, Dominik R. Bach

**Affiliations:** 1 Transdisciplinary Research Area “Life and Health”, Hertz Chair for Artificial Intelligence and Neuroscience, University of Bonn, Bonn, Germany; 2 Wellcome Centre for Human Neuroimaging, University College London, London, UK; 3 Department of Psychiatry, Psychotherapy, and Psychosomatics, University of Zurich, Zurich, Switzerland; 4 Department of Neuroscience and Biomedical Engineering, Aalto University, Espoo, Finland; 5 Department of Cognition, Emotion, and Methods in Psychology, University of Vienna, Vienna, Austria

**Publisher Correction: Behavior Research Methods**

10.3758/s13428-024-02341-3

The original online version of this article was revised: a typographical error was introduced during typesetting this article.

The first line in Table 3 was mistakenly repeated.

The original article has been corrected.

